# The Effect of Climbing Ability and Slope Inclination on Vertical Foot Loading Using a Novel Force Sensor Instrumentation System

**DOI:** 10.2478/hukin-2014-0112

**Published:** 2014-12-30

**Authors:** Jiří Baláš, Michaela Panáčková, Soňa Jandová, Andrew J. Martin, Barbora Strejcová, Ladislav Vomáčko, Jan Charousek, Darryl J. Cochrane, Mike Hamlin, Nick Draper

**Affiliations:** 1 Faculty of Physical Education and Sport, Charles University, Prague, Czech Republic.; 2 Faculty of Science, Humanities and Education, Technical University, Liberec, Czech Republic.; 3 School of Sport and Exercise, Massey University, Palmerston North, New Zealand.; 4 Faculty of Environment, Society and Design, University of Lincoln, Lincoln, New Zealand.; 5 Department of Life Sciences, University of Derby, Derby, UK.; 6 School of Sport and Physical Education, University of Canterbury, Christchurch, New Zealand.

**Keywords:** indoor climbing, vertical force, Pedar X insole, force sensor, oxygen uptake

## Abstract

The aim of the study was to assess the effects of climbing ability and slope inclination on vertical loading both in terms the forces involved and physiological responses. Five novice and six intermediate female climbers completed a climbing route at three slope inclinations (85°, 90°, and 98°). The vertical loading during the climb was assessed by force-time integral using a Novel Pedar-X insole and physiological responses via oxygen uptake and heart rate. The novice climbers had a significantly lower (p < 0.05) vertical loading on foot holds and higher oxygen uptake and heart rate compared to intermediate climbers. A significant negative correlation was identified between the force-time integral and oxygen uptake (R = −0.72), and with heart rate (R = −0.64), respectively. The time-force integral decreased across the ascents with increasing slope inclination (p < 0.001). The results indicate that more advanced ability climbers make greater use of foot holds, with associated lowering in physiological response (oxygen uptake and heart rate) across all slope inclinations.

## Introduction

Indoor climbing, like other disciplines within the rock climbing family, places demands on the muscles of the upper and lower limbs, as well as requiring core strength. Research suggests that upper limb muscle fatigue, in particular, is a significant cause of failure during ascent (Giles et al., 2006). As a consequence, indoor climbers focus their training on developing upper limb strength and endurance with an associated emphasis on static climbing positions and dynamic movements. Elite climbers are, therefore, characterized by high levels of upper body and finger flexor strength (Baláš et al., 2012; Grant et al., 1996). Although climbing is considered to be a predominantly upper body activity, proper climbing technique with effective leg work may lower the physiological demands on the musculature of upper limbs (Phillips et al., 2012).

During vertical climbing, elite climbers have demonstrated a more balanced distribution of body mass between the limbs than non-climbers (Zampagni et al., 2011). However, the force distribution is different during tripedal positions in vertical and overhanging positions (Noé, 2006). Noé et al. (2001) observed a contralateral distribution of the reaction forces for a vertical profile, whilst more homogeneous distribution occurred during ascents of overhanging climbs. Lower force values were found on foot holds and higher force on hand holds in static quadrupedal and tripedal positions during ascent of a climb with a 10° overhang (Noé et al., 2001).

Previous research based on indoor climbing reported that the further from vertical the inclination of a wall (the more overhanging) the greater the physiological demand (Mermier et al., 1997; Watts and Drobish, 1998). These responses are most likely explained by an increased physiological demand encountered in maintaining and advancing the position on the wall, leading to an increase in isometric contractions during overhanging climbing, resulting in an increase in blood pressure and heart rate (HR) without necessarily a corresponding rise in oxygen uptake (V̇O_2_) (Sheel, 2004). Heart rate and V̇O_2_ responses also differ according to the ability level of the climber, with elite climbers typically having a lower V̇O_2_ and HR when compared to lower ability level climbers on any given route (Baláš et al., 2014). These differences perhaps occur due to elite climbers having a greater climbing economy and postural control (de Moraes Bertuzzi et al., 2007).

The development of the Novel Pedar-X pressure distribution insole creates an exciting opportunity for rock climbing researchers. Such technological advances present opportunities to develop greater understanding of the sport; specifically to enable researchers to examine in more detail the nature of exercise economy in rock climbing. If the climbing style (upper limb sparing) in higher ability climbers is related to greater climbing economy, then the vertical loading on foot holds should result in lower physiological responses during climbing. Therefore, the aim of this study was to assess force loading on foot holds for novice and intermediate climbers making ascents on climbs of different slope inclination and to examine the resultant effect on the physiological demand. A second aim was to examine the nature of the relationship between the force loading and time of loading (force-time integral) with the physiological demands of a route.

## Material and Methods

### Participants

Five novice (age: 20.9 ± 0.6 years; body height: 1.64 ± 0.05 m; body mass: 57.4 ± 7.3 kg) and six intermediate (age: 25.0 ± 4.1 years; body height: 1.65 ± 0.06 m; body mass: 57.4 ± 3.4 kg) female climbers volunteered to participate in the study. The climbers were recruited from physical education students who had an interest in climbing. The novice climbers reported their RP (Red point – pre-practised ascent; YDS – Yosemite Decimal System; UIAA - Union Internationale des Associations d’ Alpinisme) climbing abilities between 5.4 – 5.7 YDS, (from 3-to 4 sport; from 4- to 5+ UIAA scales). The climbing ability of intermediate climbers ranged between 5.10b to 5.12a YDS (from 6a to 7a+ sport; from 6+ to 8+ UIAA scales) as defined by the comparison scale reported by Draper et al. (2011). The study was approved by the Ethics Committee of Charles University in Prague, Faculty of Physical Education and Sport, and all participants read and completed an informed consent form.

### Experimental Design

**Figure 1:**
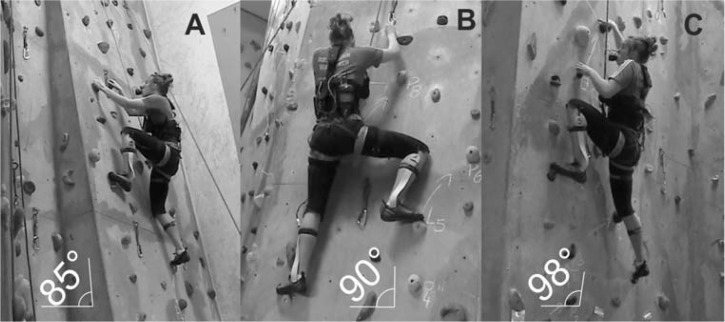
Illustration of the control system for the study with climber in the positively inclined (A), vertical (B) and overhanging (C) wall.

All climbers completed a climbing route on a wall 7.2 m high on three separate occasions with the wall angle set at (85°), vertical (90°) and overhanging (98°). The subjects climbed the same route for each inclination (trial). The climbs consisted of two ascents and two descents at a given speed of 25 movements·min^−1^. Speed of climbing was determined through pilot study work and after consultation with climbers of a variety of abilities and controlled by a digital metronome. The climbing route involved 17 hand holds/movements, but foot movements were self-selected by each climber. The total climbing time for each route was ∼ 2 min 45 s, with the order of inclination randomly assigned. Climbers had a 10 min rest interval between each trial. All climbs were performed with a top rope, where the rope passed through a top anchor between the climber and the belayer. The control system for the study is illustrated in Figure 1.

### Foot Force Analysis

The vertical reaction force under each foot was assessed using a Novel Pedar-X pressure distribution insole (Novel, Munich, Germany). Each insole contained 99 force sensors with a spatial resolution of approximately 10 mm (2 sensor/cm^2^) with a working dynamic range of 15– 600 kPa. The insole was connected to the Pedar-X box, which was attached to the waist of each participant. Pedar-X data acquisition software was used to collect and filter data and from each foot with data sampled at a frequency of 100 Hz. Before each trial, the mean level of each sensor was measured while the foot was lifted for 3–5 s and this value was used as a baseline level. A video camera (Canon ZR 960, Japan) was used to synchronize the force measurement with the record of the climbs. The sampling frequency was 25 Hz and video data was used to identify the ascent and descent phases for the force evaluation. Climbers in the study had very similar shoes sizes and all shoes were symmetric in style to control for any effect of shoe size or shape.

### Respiratory and Heart Rate Analysis

Minute ventilation (V̇_E_), oxygen uptake (V̇O_2_) and carbon dioxide production (V̇CO_2_) were measured during the climbs by a portable breath-by-breath indirect calorimetry system (MetaMax®, Cortex Biophysic, Germany). The MetaMax® was fixed onto the chest over the Pedar-X box by a harness. Before testing, the device was calibrated with a known gas mixture of 15% O_2_ and 5% CO_2_. Additionally, before each climb, auto-calibration of ambient air and volume calibration according to the manufacturer’s guidelines were performed. Volume calibration was performed using a 3L syringe. Data was averaged over 20 s intervals and the last minute from all climbing conditions was taken for analysis. The respiratory exchange ratio was computed by dividing measured V̇CO_2_ by measured V̇O_2_. Heart rate (HR) was monitored through MetaMax® using a Polar heart transmitter belt (Polar Electro OY, Finland).

### Statistical Analysis

Vertical force loading was calculated for each foot support and for the time of forceapplication. As there could have been different foot contact time with the climbing holds, the sum of force-time integral for the left and right foot was used for analysis. Force was calculated from the pressure values of all sensors and normalized to the body mass of climbers. Descriptive statistics (means and standard deviations) were used to characterize the vertical loading on the foot support and physiological responses during climbing. Repeated measures ANOVA (2 × 3) with climbing ability (novice/intermediate) as the between subject factor and inclination (85°, 90°, 98°) as the within participant factor was used to assess the effect of climbing ability and inclination on the vertical loading onto the foot support and physiological variables. In addition, Pearson product moment correlations were calculated to examine the relationship between force time integral and each of the physiological measures. An α level 0.05 was set to accept statistical significance and effect size was estimated by partial eta squared (η_p_^2^). All statistical analyses were performed using the statistical software SPSS for Windows Version 19 (Chicago, IL, USA).

## Results

Descriptive data for novice and intermediate climbers on each wall inclination are presented in Table 1. There was no significant effect for age within or across the groups so no further analyses or corrections were made for this factor. The novice climbers had lower vertical loading on the foot holds and higher physiological responses. As can be seen in Table 1, there was a significant correlation between the force-time integral and both V̇O_2_ and HR. The nature of this relationship indicated that higher vertical loading on the foot support corresponded to a lower heart rate and energy cost.

During ascent, the vertical loading on the foot support (2624 ± 411 N•kg-1•s) was significantly higher (p < 0.01) compared to descent (2488 ± 387 N•kg-1•s) at all three slope inclinations, suggesting higher arm involvement during descent.

**Figure 2:**
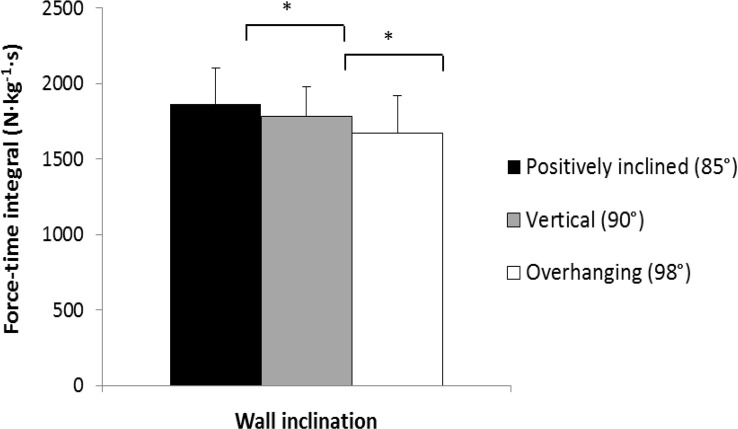
Vertical loading on the foot holds during climbing on positively inclined, vertical and overhanging walls. * indicates significant differences between inclinations.

There was a significant effect of inclination on the force-time integral on the foot support (p < 0.001; ηp2 = 0.90). For 98° inclination (overhanging), we found a 6% lower force-time integral on the foot support. Conversely, a 5% higher force-time integral was reported on the foot support at 85° (positively inclined), which corresponded to a 1% force and time integral change at a 1° wall inclination (Figure 2).

## Discussion

Our data indicated that intermediate climbers applied higher vertical loading on the foot holds than the novice climbers, which corresponded to a lower V̇O_2_ and HR response. A second finding was that vertical loading on foot holds progressively decreased with higher slope inclination.

To assess vertical loading of foot holds, we used the sum of force-time integral for left and right leg. Intermediate climbers recorded a higher force-time integral, which may be explained by the higher vertical force or longer time spent on the foot hold. Time on the foot holds should not have influenced the results because the climbing speed was held constant and the analysis of force in novice and intermediate climbers demonstrated higher mean force for both legs in intermediate climbers.

Noé et al. (2001) previously stated that during a standardized static quadrupedal position, vertical forces on the foot hold were approximately 34% lower at 10° overhanging when compared to those in a vertical position, where the overhanging position increased the vertical force on hand support by ∼ 47%. Our findings are in agreement with Noé et al.’s and confirm lower vertical loading on the foot holds during continuous climbing with progressive inclination from positively inclined to overhanging route. Additionally, the current study demonstrated that intermediate climbers applied higher vertical loading on foot holds when compared with novice climbers. This may be explained by a higher lateral centre of mass oscillation between foot holds in more experienced climbers (Zampagni et al., 2011), which is likely attained through improvedclimbing technique and movement economy for the intermediate climbers. Although we did not measure the loading on hand holds, it would seem appropriate to speculate that higher vertical loading on the foot holds, taken in conjunction with the physiological data are indicative of a lower loading on upper limbs.

Upper limb strength often represents the main limitation when climbing a difficult route and results in the climber falling because they are unable to maintain their position on a hold or to successfully execute the next move (point of exhaustion). In intermediate climbers the decreased arm involvement during climbing may be the main reason for a lower physiological response. According to de Moraes Bertuzzi et al. (2007), HR and V̇O_2_ were lower by 9 beats•min-1 and 7.3 ml•kg-1•min-1 respectively during easy climbing on a vertical route in elite compared to recreational climbers. In our study, we found mean differences between novice and intermediate climbers for HR (21 beats•min-1) and V̇O_2_ (3.3 ml•kg-1•min-1). A higher difference in the HR may be explained by individual HR max variability in climbers or by the nature of overhanging routes, which can elicit higher isometric finger flexor contractions and, therefore, HR response (Sheel, 2004).

We found a significant negative relationship between vertical loading on foot holds and V̇O_2_ (R = −0.72) and HR (R = −0.64) indicating 52% variability in V̇O_2_, and 41% in HR being associated with the force component. These findings support the notion that effective leg involvement has a direct implication on the physiological demand during climbing.

When comparing loading during ascent and descent, higher vertical loading on foot holds occurred when ascending, which implies that climbing down is more strenuous on the upper limbs than when ascending a route. However, we did not associate the physiological responses with descent and ascent because of the short time interval between ascending and descending the climb and consequently it is not possible to elucidate further the relative physiological demands of ascending and descending the route..

The main strength of the current study was the simultaneous recording of force and physiological responses during climbing. Moreover, the force analysis was undertaken in dynamic conditions which brought a picture of force distribution in a real climbing situation. The limitation of the study related to the forced speed which may have differed from the self-selected speed and therefore affected an individual’s climbing technique. However, the speed control was necessary to enable the measurement of force in this context. An additional weakness of the study relates to the small sample size which of course limits the generalizability of the results.

In conclusion, our results indicate that climbers with higher climbing ability place higher vertical loading on foot holds, which was associated with lower physiological demands (V̇O_2_ and HR). The vertical loading on the foot holds progressively decreased with higher slope inclination. Climbers should perhaps, consider the role of climbing technique in their training as it can increase the vertical loading on foot holds, significantly decreasing loading on the arms and the overall physiological demand of a climb.

## Figures and Tables

**Table 1:** Mean ± SD for vertical loading on the foot holds and an average heart rate (HR), oxygen uptake (V̇O_2_), ventilation (V̇_E_) and respiratory exchange ratio (RER) during climbing on inclined, vertical and overhanging walls. The R indicates the relationship of physiological variables to the force-time integral. * significant relationship

	*Novice climbers (N =5)*	*Intermediate climbers (N=6)*	p*,* η_p_^2^	R
	
Force Time (N·kg^−1^·s)	4485 ± 549	5635 ± 540	**p < 0.01; η_p_^2^ = 0.57**	1.00
Av. HR (beats·min^−1^)	173 ± 13	152 ± 9	**p < 0.05; η_p_^2^ = 0.53**	−0.64*
V̇O_2_ (ml·kg^−1^·min^−1^)	35.0 ± 2.1	31.7 ± 0.7	**p < 0.05; η_p_^2^ = 0.62**	−0.72*
V̇_E_ (l·min^−1^)	47.5 ± 6.4	40.0 ± 3.8	**p < 0.05; η_p_^2^ = 0.39**	−0.59
RER (V̇CO_2_ /V̇O_2_)	0.88 ± 0.03	0.83 ± 0.04	**p < 0.05; η_p_^2^ = 0.38**	−0.27

## References

[b1-jhk-44-75] Baláš J, Pecha O, Martin AJ, Cochrane D (2012). Hand-arm strength and endurance as predictors of climbing performance. Eur J Sport Sci.

[b2-jhk-44-75] Baláš J, Panáčková M, Strejcová B, Martin AJ, Cochrane DJ, Kaláb M, Kodejška J, Draper N (2014). The relationship between climbing ability and physiological responses to rock climbing. Sci World J.

[b3-jhk-44-75] de Moraes Bertuzzi RC, Franchini E, Kokubun E, Peduti Dal Molin Kiss MA (2007). Energy system contributions in indoor rock climbing. Eur J Appl Physiol.

[b4-jhk-44-75] Draper N, Couceiro Canalejo J, Fryer S, Dickson T, Winter D, Ellis G, Hamlin M, Shearman J, North C (2011). Reporting climbing grades and grouping categories for rock climbing. Isokinet Exerc Sci.

[b5-jhk-44-75] Giles LV, Rhodes EC, Taunton JE (2006). The Physiology of rock climbing. Sports Med.

[b6-jhk-44-75] Grant S, Hynes V, Whittaker A, Aitchison T (1996). Anthropometric, strength, endurance and flexibility characteristics of elite and recreational climbers. J Sport Sci.

[b7-jhk-44-75] Mermier CM, Robergs RA, McMinn SM, Heyward VH (1997). Energy expenditure and physiological responses during indoor rock climbing. Br J Sport Med.

[b8-jhk-44-75] Noé F (2006). Modifications of anticipatory postural adjustments in a rock climbing task: The effect of supporting wall inclination. J Electromyogr Kines.

[b9-jhk-44-75] Noé F, Quaine F, Martin L (2001). Influence of steep gradient supporting walls in rock climbing: biomechanical analysis. Gait Posture.

[b10-jhk-44-75] Phillips KC, Sassaman JM, Smoliga JM (2012). Optimizing rock climbing performance through sport-specific strength and conditioning. Strength Cond J.

[b11-jhk-44-75] Sheel AW (2004). Physiology of sport rock climbing. Br J Sport Med.

[b12-jhk-44-75] Watts PB, Drobish KM (1998). Physiological responses to simulated rock climbing at different angles. Med Sci Sport Exer.

[b13-jhk-44-75] Zampagni ML, Brigadoi S, Schena F, Tosi P, Ivanenko YP (2011). Idiosyncratic control of the center of mass in expert climbers. Scand J Med Sci Sport.

